# Lung Ultrasound (LUS) and neonatal respiratory distress

**DOI:** 10.1186/1824-7288-41-S2-A13

**Published:** 2015-09-30

**Authors:** Luigi Cattarossi

**Affiliations:** 1 SOC di Patologia Neonatale. Azienda Ospedaliero Universitaria S. Maria della Misericordia. Udine-Italy

## Introduction

Neonatal lung diseases are often a diagnostic dilemma for the clinician due to the low sensitivity and specificity of clinical signs and symptoms. In the last decade of the previous century the use of ultrasound in the diagnostic work-up of adult respiratory diseases became widely used [1,2]. The purpose of this paper is to update the knowledges on LUS in the most common neonatal respiratory diseases [3,4].

## Materials and methods

A high resolution linear probe 10 MHz or more is used for lung examination. Longitudinal and transversal sections of the anterior, lateral and posterior wall are obtained. In a normal lung the pleura appears as a regular echogenic line moving during respiration. Beyond the pleura the change in acoustic impedance at the pleura-lung interface results in horizontal artifacts, defined as A-lines [5]. Vertically oriented artifacts, called B-lines, indicate an abnormality amount of fluid in the interstitial or alveolar compartment [1].

## Results

Respiratory Distress Syndrome (RDS)

RDS diagnosis is based on the presence of echographic white lung without spared areas, thickened pleural line [4] . The LUS appearance immediately after administration of exogenous surfactant does not change [6].

Transient Tachypnea of the Newborn (TTN)

TTN has normal pleural line and pleural sliding, with compact B-lines in the inferior pulmonary fields and few B-lines in the superior fields [3], or bilateral “wet lung” defined as presence of numerous non-compact B-lines.

Meconium Aspiration Syndrome (MAS)

MAS shows a picture of coalescent B-lines and subpleural consolidations along with few spared areas. Subpleural consolidation distribution is irregular and may be more evident in one side.

Pneumothorax

LUS signs of pneumothorax are absence of lung sliding, absence of B-lines and evidence of “lung point”. Air between parietal and visceral pleura does not allow to see the movement of the visceral pleura on the parietal pleura and the B-lines that originate from visceral pleura, Lung point when present has a sensitivity and specificity of 100% [2]. Can be seen when the partially collapsed lung inflates and parietal and visceral pleura are in contact and lung sliding is again evident.

## Conclusions

In neonatal age the use of LUS is becoming a new and reliable tool in the hand of the clinician.

LUS does not substitute chest X-ray, but can reduce its use with benefits in terms of irradiation risk [7]. The use of LUS in the clinical practice is a promising and already well established entity in neonatal age.
